# Progress in Otology

**Published:** 1900-06-02

**Authors:** 


### Progress in Otology.

**Otitis Media.**—Marcel Lermoyez,[1] as the result of a number of observations, is firmly convinced that otitis media is often the result of contagion, not of an obvious contagion, but of one which is insidious and easily escapes observation. He briefly describes seven, out of a total number of 20 cases of acute otitis media treated by him in two years, in which inflammation of the same type was apparently developed as a result of contagion. The patients infected one another by the clasical naso-tubal route; they commenced necessarily by having a coryza, but this was so attenuated as to pass unperceived. The cases were of secondary otitis. The observations tended to prove that "given the first patient affected with influenza complicated with otitis, any other influenzal patient put in contact with him will run a great risk of acquiring this otitic complication." Statistics are given showing the frequency of secondary otitis in hospital, its rarity in private practice. Isolation of patients, especially children, with acute otitis is therefore strongly recommended.

Discussing the therapy of otitis media, Goldstein[1] divided the subject into two sections: (1) Where the membrana tympani was intact; and (2) where perforations of the tympanic membrane existed. The catarrhal and non-suppurative forms came in the first group, the suppurative form in the latter. The therapy of the catarrhal form had varied much in recent years. It included such procedures as inflation of the tympanum with the air-bag of Politzer, or with the Eustachian catheter; injections of fluid medicaments in an aqueous or an oleaginous menstruum; injection of pepsin through the drum membrane with a hypodermic syringe; the introduction of vapours of ammonium chloride, chloroform, &c.; the use of the pressure probe of Lucae, or of the Eustachian bougie; and massage of the drum membrane by the hand-masseur or the electric masseur engine. The results were only moderately good. As atrophic rhinitis might be considered the sequel of the hypertrophic form, so sclerotic or adhesive otitis might be regarded as the sequel of hypertrophic otitis. Goldstein had used purified petroleum as a base for his medications, and had found iodine, menthol, carbolic acid, eucalyptus, &c., the most useful agents. If the Eustachian tube was free, as shown by the auscultation tube, the fluid was injected through the Eustachian tube. Where that offered obstruction, he had used continued vapour inflations with good results. For this purpose he employed a hand-nebuliser with a glass nasal tip. Following the vaporisation or injection, he used the aural masseur. He believed that the treatment of chronic purulent otitis by irrigation through the meatus was contra-indicated where there was a large perforation, and ready entrance of the fluid into the tympanum was provided. Infection of the attic or antrum might be caused by driving infectious material into them with the syringe. Again, it was irritation of the sodden mucous membrane by fluids which led to the growth of polypi which the addition of aqueous fluids aggravated. The indiscriminating use of the nasal douche frequently led to infection of the tympanum. H. A. Alderton[2] advocates strongly the use of syringing in these cases, at least a pint of liquid every two hours being recommended. An antiseptic is not necessary if the canal is thoroughly dried, as the solution never reaches the middle ear. He regards the chances of forcing infected material into the mastoid cells as not worthy of consideration in contrast with the benefits derived. In all cases of otitis media in which the mastoid did not show any traces of inflammation during life pus was found post-mortem in the mastoid cells. Powders were unsuitable in acute suppuration; they tended to pack in front of the drum and impeded drainage, and did not reach the inflamed surface. Stetter[3] supports Lucae in the latter's statement that the general symptoms calling for the radical operation for otitis are marked dizziness with disturbances in gait, nausea, and vomiting; while the local symptoms pointing that way have to do with the duration of the disease, the condition of the post-aural region, the pain, the facial paralysis, and the nature and quality of the discharge. The value of iodide of potash in rendering the thick tenacious pus more fluid so that the ear can be properly cleansed is pointed out. Also Hang's chinolin-naphthol gauze as a packing is recommended. It is very soft and of open mesh, and can be packed even into the middle ear itself. Pure trichloracetic acid is applied to prominent granulations; for overcoming the fœtid and stinking condition of the discharge menthoxol with an equal part of water is praised. In contact with pus it gives off oxygen, and leaves behind menthol dissolved in alcohol. It has been very successful. Urban Pritchard[4] has contributed a paper on the antiseptic purification of the meatus for operations, and as a treatment in middle ear suppurations. The treatment is specially adapted to cases such as the following: (1) When there is no suppuration and the membrana tympani is intact, for it allows of operations without infection of deeper parts. (2) When there is perforation and suppuration already present. It is not possible to produce absolute asepticism in the very septic cases. The mode of procedure in the first class—*e.g.*, in preparing for removal of an exostosis, or for any operation, is to syringe well with 1 in 40 carbolic, and then mop out with 1 in 20 until the parts are quite clean, so far as the eye can judge. This is done an hour before operation. The whole auricle is then well scrubbed with 1 in 20, and then the meatus lightly packed with a strip of double cyanide gauze, wrung out of a 1 in 20 or 40 carbolic to get rid of the irritating soluble cyanides, and a pad of similar gauze is fixed over the auricle. The operation is then conducted with all the antiseptic precautions used in ordinary surgery, and if anything needs removing it is done by syringing with 1 in 40 carbolic, the syringe being perfectly purified first. Ordinary antiseptic dressings are used after the operation. In paracentesis in acute otitis suppuration may often be altogether prevented if it has not already occurred, which is frequently the case. In cases where suppuration already exists, *e.g.*, where polypi have to be removed, or carious spots curetted, the ear should be syringed out with 1 in 40 carbolic once or twice a day for a week before the operation. The syringing must be repeated after the operation, when frequently epidermic caseous masses will be dislodged. If the case is very septic the strip of gauze should be tipped with iodoform powder. Granulations and polypi treated in this way yield often excellent results. The gauze packings have to be repeated once a day to once in two, three, or more days according to the conditions present. In chronic otorrhœa, even after the discharge has ceased, it is well to continue the dressing of gauze, using a superficial and a deep piece, without a bandage, so that if the patient works out the superficial piece the other still remains undisturbed. Lysol or perchloride of mercury might be substituted for carbolic in some cases. E. B. Gleason[5] contributes a paper on the silver salts in the treatment of chronic suppuration of the middle ear. The use of nitrate of silver in these cases had not been remarkably successful, as the irritating effects had overshadowed the antiseptic and astringent properties. Certain organic salts of silver have been stated to act as astringents for mucous membranes without being in the least irritating. This led Gleason to think they might prove very useful in treating prolonged otorrhœas in which the attic and probably the mastoid autrum were involved. Protargol was therefore tried in a 5 per cent. solution injected as high up into the attic as possible by means of a Blake's cannula. Then the parts were massaged with Siegle's pneumatic speculum in order to force the solution into more distant parts than could be reached with the syringe. Before this the middle ear had been cleansed and dried. Four attic cases are detailed, in three of which the treatment was highly successful. The solution is quite unirritating to the middle-ear mucous membrane.

[1] Vide Jour. Laryng., Rhinol, and Otol., Dec., 1899. [2] Laryngoscope, Jan., 1900. [3] Vide Laryngoscope, Jan., 1900. [4] Jour. Laryng., Rhinol. and Otol., Mar., 1900. [5] Laryngoscope, Mar., 1900.

